# Supplementary figures and images for: Three-dimensional, printed water-filtration system for economical, on-site arsenic removal
Source: PLoS One. 2020 Apr 24;15(4):e0231475. doi: 10.1371/journal.pone.0231475 (PMC7182265; doi:10.1371/journal.pone.0231475)

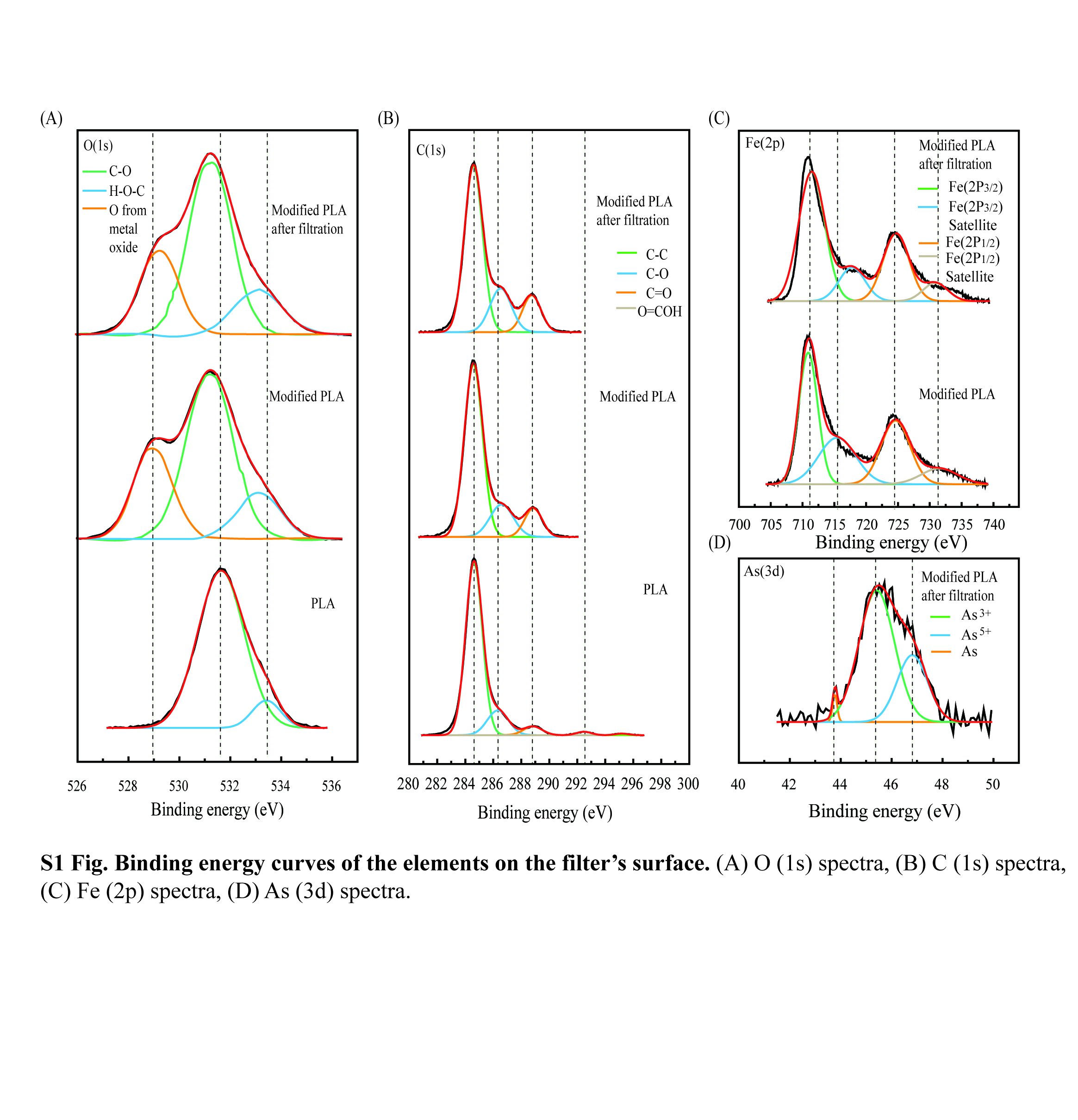

Supplement: S1 Fig — Binding energy curves of the elements on the filter’s surface: (A) O (1s) spectra, (B) C (1s) spectra, (C) Fe (2p) spectra, (D) As (3d) spectra. (TIF) [file pone.0231475.s001.tif]

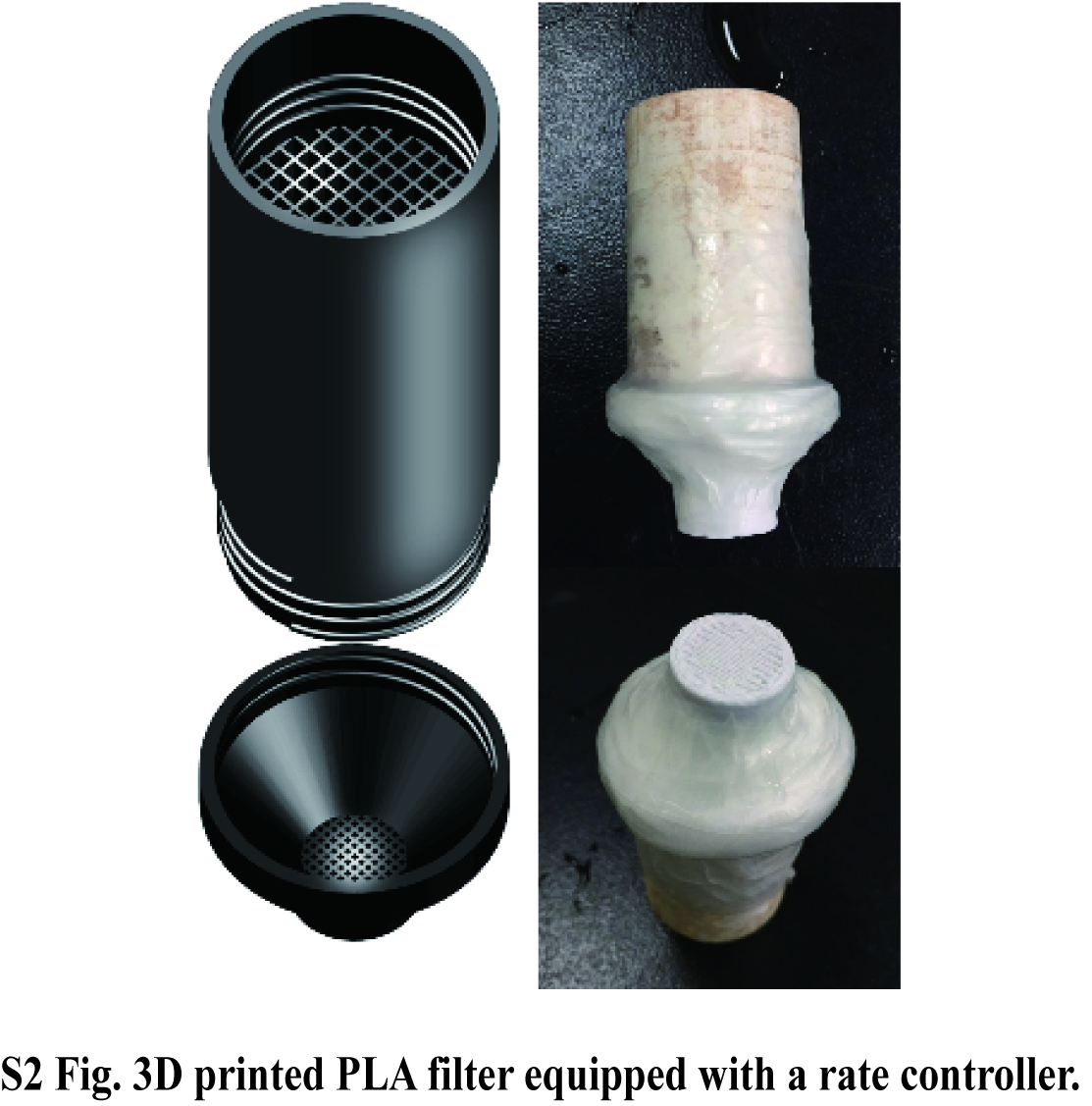

Supplement: S2 Fig — (TIF) [file pone.0231475.s002.tif]
