# Supplementary material for: Three-dimensional, printed water-filtration system for economical, on-site arsenic removal
Source: PLoS One. 2020 Apr 24;15(4):e0231475. doi: 10.1371/journal.pone.0231475 (PMC7182265; doi:10.1371/journal.pone.0231475)
Supplement: S1 Table — (DOCX) [file pone.0231475.s003.docx]

**S1 Table. Kinetic parameters for As (III) removal by filters with four different channel widths**.

| Channel width (mm) | 4.0 | 1.8 | 1.0 | 0.8 |
| --- | --- | --- | --- | --- |
| $k_{2}$ (gmg^-1^mL^-1^) | 97.26 | 470.44 | 1733.94 | 2913.97 |
| $q_{e}$ (mgg^-1^) | 6.485 | 6.046 | 5.845 | 5.814 |
| R^2^ | 0.9773 | 0.9986 | 0.9990 | 0.9999 |

The parameters were estimated by using the pseudo-second order equation.
