# Supplementary material for: Three-dimensional, printed water-filtration system for economical, on-site arsenic removal
Source: PLoS One. 2020 Apr 24;15(4):e0231475. doi: 10.1371/journal.pone.0231475 (PMC7182265; doi:10.1371/journal.pone.0231475)
Supplement: S2 Table — (DOCX) [file pone.0231475.s004.docx]

**S2 Table.** **Isotherm parameters for As (III) removal by filters with four different channel widths.**

| Channel width (mm) | 4.0 | 1.8 | 1.0 | 0.8 |
| --- | --- | --- | --- | --- |
| $q_{max} (mg/g)$ | 16.6 | 23.8 | 129.9 | 125.0 |
| $b (L{mg}^{-1})$ | 1.275 | 0.921 | 0.166 | 0.181 |
| R^2^ | 0.9305 | 0.9164 | 0.9036 | 0.9238 |

The parameters were estimated by using the Langmuir isotherm model.
