# Supplementary material for: Three-dimensional, printed water-filtration system for economical, on-site arsenic removal
Source: PLoS One. 2020 Apr 24;15(4):e0231475. doi: 10.1371/journal.pone.0231475 (PMC7182265; doi:10.1371/journal.pone.0231475)
Supplement: S3 Table — (DOCX) [file pone.0231475.s005.docx]

**S3 Table. Reusability of 3D-printed filter.** The filter with a channel width of 0.8 mm was tested using 15 mL of model contaminated water with an As (III) initial concentration of 20 mg/L.

| Cycle number | Concentration after filtration* | Percent removal of arsenic (%) |
| --- | --- | --- |
| 1 | 0.77 | 96.2 |
| 2 | 2.73 | 86.4 |
| 3 | 4.56 | 77.2 |

y=0.475x + 0.008

R^2^= 0.996

*Calibration curve of As (III) for the determination of As (III) contamination after filtration
