# Supplementary material for: Three-dimensional, printed water-filtration system for economical, on-site arsenic removal
Source: PLoS One. 2020 Apr 24;15(4):e0231475. doi: 10.1371/journal.pone.0231475 (PMC7182265; doi:10.1371/journal.pone.0231475)
Supplement: S4 Table — (DOCX) [file pone.0231475.s006.docx]

**S4 Table. Binding energies, percentage of total peak area, and assignment of atoms of XPS spectra of O (1s), C (1s), Fe (2p) and As (3d) from PLA, iron (III) oxide** **modified PLA filter, and iron (III) modified PLA filter after filtration.**

| **Sample** | **BE (eV)** | **% of total area** | **Assignment of atoms** |
| --- | --- | --- | --- |
| O (1s) | | | |
| PLA | 531.63 | 90.36 | C-O |
|  | 533.41 | 9.63 | H-O-C |
| Iron (III) oxide modified PLA | 529.41 | 26.83 | Metal oxide |
|  | 531.25 | 58.25 | C-O |
|  | 532.85 | 14.91 | H-O-C |
| Modified PLA filter after filtration | 529.63 | 25.35 | Metal oxide |
|  | 531.26 | 58.11 | C-O |
|  | 532.80 | 16.53 | H-O-C |
| C (1s) | | | |
| PLA | 284.62 | 79.33 | C-C |
|  | 286.32 | 14.26 | C-O-C |
|  | 288.83 | 4.47 | O-C=O |
|  | 292.48 | 1.37 | O=COH |
| Iron (III) oxide modified PLA | 284.58 | 71.30 | C-C |
|  | 286.60 | 17.21 | C-O-C |
|  | 288.86 | 11.49 | O-C=O |
| Modified PLA filter after filtration | 284.58 | 65.20 | C-C |
|  | 286.53 | 20.18 | C-O-C |
|  | 288.75 | 14.62 | O-C=O |
| Fe (2p) | | | |
| Iron (III) oxide modified PLA | 710.9 | 37.55 | Fe(2p_3/2_) |
|  | 715.45 | 26.08 | Fe(2p_3/2_) satellite |
|  | 724.61 | 27.72 | Fe(2p1_/2_) |
|  | 730.88 | 8.65 | Fe(2p1_/2_) satellite |
| Modified PLA filter after filtration | 711.44 | 52.13 | Fe(2p_3/2_) |
|  | 717.79 | 13.58 | Fe(2p_3/2_) satellite |
|  | 724.72 | 27.30 | Fe(2p1_/2_) |
|  | 731.51 | 6.98 | Fe(2p1_/2_) satellite |
| As (3d) | | | |
| Modified PLA filter after filtration | 43.77 | 1.83 | As satellite |
|  | 45.42 | 68.36 | As^3+^ |
|  | 46.81 | 29.81 | As^5+^ |
